# Supplementary figures and images for: Genomic Epidemiology of Candida auris in Qatar Reveals Hospital Transmission Dynamics and a South Asian Origin
Source: J Fungi (Basel). 2021 Mar 23;7(3):240. doi: 10.3390/jof7030240 (PMC8004815; doi:10.3390/jof7030240)

Fig. S1

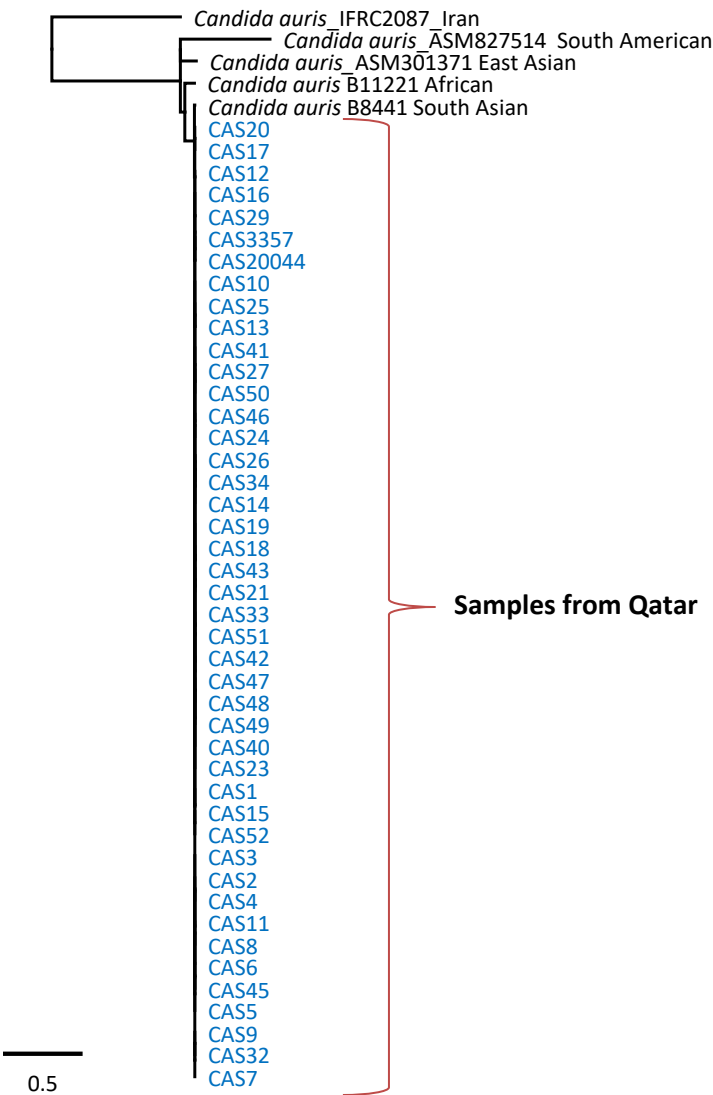

Supplement: Supplementary file 1 [file jof-07-00240-s001.zip › cas-FigS1-revised.pdf]
